# A Rare Case of Necrotizing Fasciitis in a Female With Diabetes Mellitus

**DOI:** 10.7759/cureus.64905

**Published:** 2024-07-19

**Authors:** Chalent Alexakis, Konstantinos Zacharis, Spyridon Chondros, Stavros Kravvaritis, Theodoros Charitos

**Affiliations:** 1 Department of Obstetrics and Gynaecology, General Hospital of Lamia, Lamia, GRC

**Keywords:** type 1 diabetes mellitus, multidisciplinary decision-making, necrotizing fascitis, obesity and diabetes, fournier gangrene

## Abstract

Fournier's gangrene (FG) is a relatively rare yet profoundly severe disease. It predominantly affects males; however, mortality rates are comparatively elevated in females. It is a rapidly spreading, life-threatening necrotizing fasciitis that can affect all parts of the body but primarily targets the genital region and the perineum. The clinical presentation is highly characteristic of the disease and is often sufficient for reaching a definitive diagnosis. Common risk factors for the development of this condition include diabetes mellitus (DM), obesity, trauma, alcoholism, smoking, arterial hypertension (which predisposes to obstructive endarteritis), and immunosuppressive disorders, such as HIV and cancer. Prompt diagnosis and treatment are imperative for the prognosis and survival of patients. Herein, we present a case of a 33-year-old woman with a medical history of type 1 diabetes mellitus (treated with insulin), arterial hypertension, and obesity. She presented with pain and swelling in the external genitalia (right labia majora), which later progressed to severe necrotizing fasciitis. The patient underwent surgical debridement and drainage, along with intensive medical therapy.

## Introduction

While Baurienne described a case of scrotal gangrene in a 45-year-old butcher of the army in 1764, the disease came to be known as Fournier's gangrene (FG) after the venereologist Jean Alfred Fournier. In his published work in 1883, Fournier documented a case of fulminant gangrene characterized by a sudden onset, unknown etiology, and rapid necrosis of the scrotal and penile skin in a series of five young, otherwise healthy men [1]. This condition is a rapidly developing gangrenous fascitis (necrotizing fascitis) located in the external genitalia, the perineum, and the anus area. The basic pathology of the disease is the destruction of the subcutaneous fatty layer, where nerves and vessels are involved, as well as the superficial and deep fascia. It is accompanied by the thrombosis of feeding vessels and leads to necrosis of the skin and the subcutaneous tissue. It manifests with severe toxicity and leads to multiple organ failure [2]. As a general rule, it affects men, but it can also be seen in women and even children. Although the precise ratio of male-to-female incidence in the disease is not established, a study conducted in the United States revealed a male-to-female ratio of 40:1 within their examined population [3].

Necrotizing fasciitis is a relatively rare infection with a rather high mortality rate of >20% that involves the muscle fascia and the subcutaneous tissue [4]. All parts of the body can be affected, with a higher prevalence in the genital region and the perineum [5]. It is a sympiotic polymicrobial infection that is caused by aerobic and anaerobic microbia, which constitute the normal flora of the respective region and originate from the lower gastrointestinal region (30-50%, e.g., perianal abscess), urinary (20-40%, e.g., urinary tract infections and catheterization-related urethral traumas), and skin (20%, e.g., perineal abscess and allergic reactions) [6]. Patients with diabetes mellitus and immunosuppression (chemotherapy and chronic use of corticosteroids), alcoholics with chronic renal failure, and individuals with pathological obesity, atherosclerosis, peripheral arteriopathy, leukemia or other malignancy, hepatic diseases, and pulmonary diseases with systemic disorders are predisposed to develop FG [7]. Although this pathological condition is known as fulminant, with symptoms of sudden pain in the perineum, accompanied by signs of inflammation, very often, it has a gradual evolution. Usually, there is a latent period of two to seven days before the onset of symptoms [8]. Itching, pain, and general discomfort gradually worsen in this period before the admission to the hospital. The most common symptomatology of FG includes scrotal swelling, hyperemia, pain and itching of the affected area, tissue crepitus, and higher fever [5]. More specifically, it has been found that it clinically presents as perianal or periscrotal pain, tachycardia, purulent discharge from the perineum, cramping, and fever [8]. The crepitus is due to the presence of air in soft tissues and is found during the clinical examination by palpating the affected area. It represents insoluble gas, produced by anaerobic bacteria, and is mainly composed of nitrogen, hydrogen, nitrous oxide, and sulfuric acid [9]. The presence of air can be demonstrated imaginably even before its clinical presence. Systemic symptoms and laboratory findings include leukocytosis or leukopenia, tachycardia, dehydration, thrombocytopenia, anemia, hyperglycemia, and hypocalcemia [4].

## Case presentation

**Figure 1:**
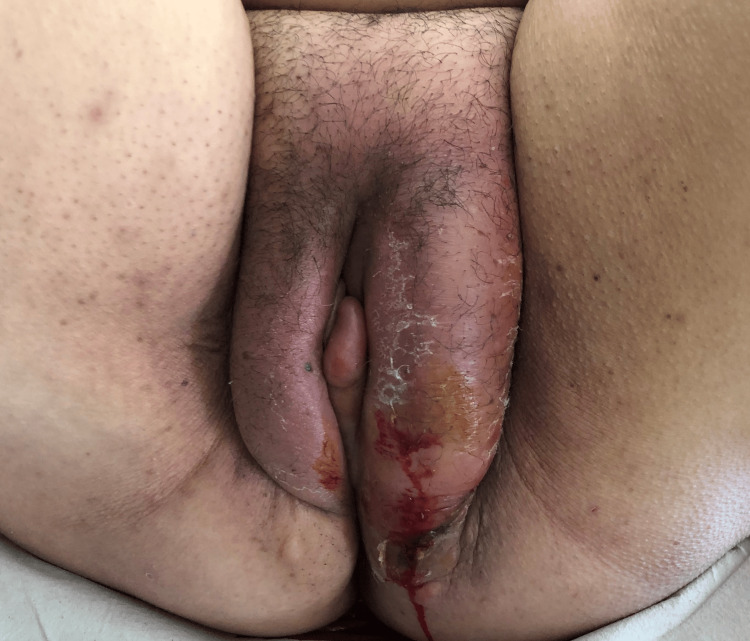
Patient presentation in the emergency department. Redness and edema in the left labia majora.

**Figure 2:**
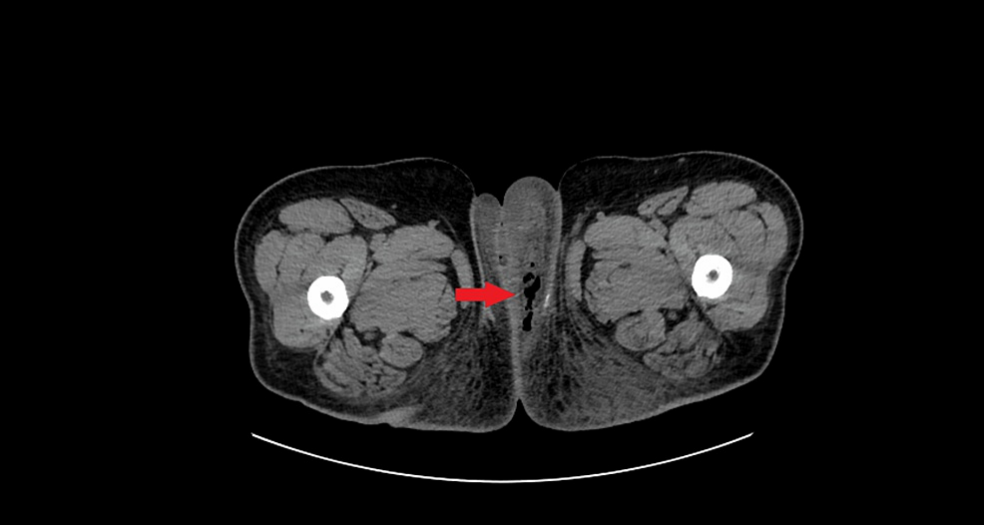
CT scan of the lower abdomen. The arrow points to the presence of free air in the subcutaneous tissue.

A 34-year-old female patient presented to the emergency department with pain in the external genital region that started 18 hours ago. The patient had a history of obesity (body mass index (BMI): 34), arterial hypertension, and type 1 diabetes mellitus treated with insulin (glargine-lispro). The clinical examination revealed severe swelling and redness in the right labia majora, with the area being extremely sensitive even to superficial palpation (Figure 1). The complete blood count showed neutrophilic leukocytosis (white blood cell count (WBC): 15,900 K/μL, neutrophils (NEU): 80.7%), C-reactive protein (CRP) was 193.8 mg/L, and HbA1c was 12.1% (Table 1). A computed tomography (CT) scan of the abdomen and pelvis with intravenous (IV) contrast was ordered immediately. The scan, seen in Figure 2, indicated a soft tissue infection sparing the muscle and fascia, with extensive subcutaneous emphysema in the subcutaneous tissue of the right labia, leading to a diagnosis of FG. The depth of the infection was found to be 3-4 cm from the skin.

**Table 1:** Laboratory values upon the patient's arrival in the emergency department

Laboratory exam	Upon presentation in the emergency department
Leukocytes (4.5–11.0 × 10^3^/μL)	15,900 K/μL
Neutrophils (2.00-6.90 × 10^3^/μL)	80.7%
C-reactive protein (CRP) (<5.0 mg/L)	193.8 mg/L
Hba1c (<5%)	12.1%

A multidisciplinary team discussion resulted in an immediate decision for wound debridement of the right labia. After induction of spinal anesthesia, the patient was placed in the lithotomy position. Severe edema occupied the entire right labia. An incision was made vertically from 12 to 6 o'clock. A culture was obtained. The pus was drained, and any remaining loculi were broken with finger manipulation. Extensive washing of the cavity was performed using hydrogen peroxide and betadine solution. A Penrose drain tube was placed in the cavity, and an aseptic dressing was applied. Empirical treatment with IV piperacillin/tazobactam and daptomycin was started. The pus culture identified *Pseudomonas aeruginosa*, *Klebsiella pneumoniae*, *Streptococcus agalactiae*, *Enterococcus faecium*, and *Candida glabrata*. According to the antibiogram, the patient was administered meropenem, tigecycline, and daptomycin.

After 10 days, the patient was discharged from the hospital and was advised to strictly monitor her blood glucose, seek medical help regarding her blood pressure, and maintain proper hygiene in the affected area. The wound was left to heal by secondary intention. She was followed weekly for four weeks and had no further complications.

## Discussion

The first description of the disease was provided by Fournier in 1883. It involved five otherwise healthy young men who presented with sudden gangrene of the scrotum of unknown etiology [10]. In recent years, the characteristics of FG have changed significantly. It is no longer considered idiopathic in most cases, as a clear causative factor can usually be identified. In addition, the disease now affects a wider age range, and its onset appears to be more insidious compared to the past [3]. The main foci of infection are found in the skin, colon, rectum, anus, and lower urinary tract. Most of the time, there is a clear etiological factor responsible for the manifestation of the disease, such as an infection or trauma of the rectal area or urinary system. The most common cause is believed to be an abscess of the perianal area and rectal and trans sphincteric abscess in particular when these are not diagnosed and treated in time [6]. The infection is polymicrobial and most often due to aerobic and anaerobic microbes. It is believed that there is a synergistic action of the microbes that cause FG. Even if anaerobic microbes are not isolated in the cultures, their presence and participation in the infection should never be ruled out. The most frequently isolated microbes with cultures are *Escherichia coli*, *Bacteroides*, and *Streptococcus* [10]. In the literature, there are cases of FG caused by *Candida* as the primary organism [11].

FG manifests with pain usually in the perineum, and swelling is found in the affected area. The skin of the area may be normal, blistered, or ecchymotic or have the characteristic black color of gangrene. The patient may present with fever, tachycardia, and a drop in blood pressure, signs indicative of a decrease in circulating blood volume due to septic shock. Symptoms from the various symptoms are disproportionate compared to the size of the visible infection. Laboratory testing shows leukocytosis, anemia, hypoalbuminemia, increased creatinine, electrolyte disturbances, and hypocalcemia [8,10]. Various imaging techniques may help to determine the extent of the infection. A simple X-ray of the abdomen and pelvis can show the presence of air in the subcutaneous tissue before crepitus is palpable [12]. Moreover, it can reveal the existence of some pathological conditions in the abdomen. In gangrene localized to the perineum, air is found in 90% of cases. The ultrasound of the scrotum, in addition to establishing the existence of air in the subcutaneous tissue, helps in the differential diagnosis of various conditions that cause acute pain in the scrotum, such as testicular rupture, epididymitis, torsion of the testicle, hematoma, abscess, and tumor. In FG, the testes appear normal [12,13]. CT of the pelvis and perineum can show the extent of the infection and the presence of subcutaneous emphysema. It is also particularly useful when the focus of infection is located in the peritoneal cavity or retroperitoneally [12]. MRI more accurately determines the anatomical sites where the infection has spread [12].

FG requires immediate surgical intervention. The immediate administration of broad-spectrum antibiotics and hemodynamic stabilization of the patient if he shows signs of septic shock are considered mandatory actions until the patient is taken to the operating room. Sometimes, it is necessary to restore the blood volume by administering crystalloid solutions, colloid solutions, or even blood. Antibiotics administered empirically until culture results are obtained must be active against gram-positive and gram-negative microbes and against anaerobic microbes. Specifically, they must be highly effective against *Streptococci*, *Staphylococci*, coliforms, *Pseudomonads*, *Bacteroides*, and *Clostridia*. Tetanus immunization with tetanus toxoid is also recommended [10]. The skin manifestations of FG are only the tip of the iceberg. The extent of the infection cannot be judged by the simple examination of the superficial tissues. The surgeon should be prepared that he may be involved in a major surgery. The necessary surgical debridement can only be done in the operating room under general anesthesia and with the presence of all the medical and nursing staff involved in any major surgery. Surgical exploration of the affected areas by the most experienced surgeon and wide resection of all necrotic tissues or tissues of questionable viability and adequate drainage of all fascial planes and empty anatomical spaces and compartments are urgently required regardless of the large resulting tissue deficit. Surgical debridement proceeds until all deep and apparently healthy tissues are exposed [14].

## Conclusions

The correct treatment of FG is multidisciplinary, which includes initial and undiagnosed diagnosis, extensive surgical debridement, targeted antibiotic therapy, correct subsequent wound delineation, and, in some selected patients, colostomy and suprapubic catheterization. At least in the initial stages, close monitoring in an intensive care unit for monitoring and follow-up of laboratory parameters are mandatory, followed by the long road of healing and plastic restoration. Given the rarity of FG cases in women, it is imperative that each diagnosed case be reported and their treatment documented to improve future clinical approaches.
